# Blood volume reduction due to rapid plasma loss after birth in preterm piglets

**DOI:** 10.1038/s41390-024-03252-x

**Published:** 2024-05-21

**Authors:** Tam M. T. Nguyen, Holly Morwood, Bhavisha A. Bakrania, Stephanie M. Miller, Stella T. Bjorkman, Barbara E. Lingwood, Ian M. R. Wright, Yvonne A. Eiby

**Affiliations:** 1 Perinatal Research Centre and UQ Centre for Clinical Research, Faculty of Medicine, The University of Queensland, Brisbane, QLD Australia; 2 Department of Neonatology, Royal Brisbane and Women’s Hospital, Brisbane, QLD Australia; 3 Australian Institute of Tropical Health and Medicine, The College of Medicine and Dentistry, James Cook University, Cairns, QLD Australia

## Abstract

**Background:**

Understanding changes in blood volume after preterm birth is critical to preventing cardiovascular deterioration in preterm infants. The aims were to determine if blood volume is higher in preterm than term piglets and if blood volume changes in the hours after birth.

**Methods:**

Paired blood volume measurements were conducted in preterm piglets (98/115d gestation, ~28wk gestation infant) at 0.5–5 h (*n* = 12), 0.5-9 h (*n* = 44) and 5–11 h (*n* = 7) after birth, and in a term cohort at 0.5–9 h (*n* = 40) while under intensive care.

**Results:**

At 30 min after birth, blood volume was significantly lower in preterm piglets compared to term piglets. By 9 h after birth, blood volume had reduced by 18% in preterm piglets and 13% in term piglets. By 5–9 h after birth, preterm piglets had significantly lower blood volumes than at term (61 ± 10 vs. 76 ± 11 mL/kg).

**Conclusions:**

In contrast to clinical resources, preterm piglets have a lower blood volume than at term. Substantial reductions in blood volume after birth leave some preterm piglets hypovolemic. If this also occurs in preterm infants, this may have important clinical consequences. Modern studies of blood volume changes after birth are essential for improving preterm outcomes.

**Impact:**

- Preterm piglets do not have a higher blood volume than their term counterparts, in contrast to current clinical estimates.
- Rapid reduction in blood volume after birth leads to hypovolemia in some preterm piglets.
- There is a critical need to understand blood volume changes after birth in preterm infants in order to improve clinical management of blood volume.

## Introduction

Extremely preterm infants (<28wk gestation) are at high risk of poor neurodevelopmental outcomes.^1,2^ Deterioration of cardiovascular function is a major contributor to this risk.^3^ but interventions to support blood volume are ineffective at improving these outcomes.^4^

There are limited data on blood volume at birth, and how it changes in the hours after birth. It is widely stated that blood volume is higher in preterm infants than at term and early studies report substantial reductions in blood volume in the minutes/hours after birth.^5,6^ However, the timing and extent of these changes under modern clinical care is unknown, and early studies used flawed methodology.^7^ More recent studies demonstrate that small plasma tracers rapidly leak from the preterm circulation, resulting in overestimation of blood volume in preterm infants.^8,9^ A few studies have used methods that overcome this bias, by either using large tracers or multiple timed samples with back extrapolation to the time of injection, and these studies report similar blood volumes in term and preterm infants (71-78 ml/kg).^10,11^ There is limited information from modern cohorts on how blood volume changes across the first day of life and its role in cardiovascular deterioration. An understanding of preterm blood volume is critical to improving clinical management of preterm cardiovascular function.

Using an established pre-clinical model,^12^ this study aims to determine if blood volume in the hours after birth is (1) higher in preterm piglets than at term, and (2) increases or decreases in the hours after birth.

## Methods

### Animals

This project was approved by the University of Queensland Animal Ethics Committee (UQCCR/534/19, UQCCR/060/12/NHMRC) in accordance with the Australian Code of Practice for the Care and Use of Animals for Scientific Purposes (version 8, 2013) prior to commencement of any experiments. Pregnant sows were sourced from commercial piggeries in south-east Queensland, Australia. Term piglets (114/115d gestation) and preterm piglets (98/115d gestation) were delivered by caesarean section. Preterm piglets at 98d gestation are developmentally similar to extremely preterm infants born at 28 weeks gestation.^12^ At 48 and 24 h before delivery, all preterm piglets received maternal glucocorticoid treatment (IM, betamethasone sodium phosphate 0.13 mg/kg and betamethasone acetate 0.1 mg/kg; Celestone Chronodose, Schering-Plough, Kenilworth, NJ), consistent with standard clinical practice in women threatening preterm labour. Sows were premedicated with Stresnil (Azaperone 40 mg/mL; 1.2–1.6 mg/kg IM) and ketamine (Ketamil 2.5–2.8 mg/kg IM; Troy Laboratories, AUS) 30 min prior to anaesthesia, then anesthetised with Alfaxalone (Alfaxane 0.5–2 mg/kg IV ear vein) or propofol (Propofol Sandoz; 40 mg IV, q10sec until onset, 2–2.5 mg/kg to effect). Following induction, sows were intubated using a 12–14 mm cuffed endotracheal tube and anaesthesia was maintained with 2% isoflurane (Attane) in O_2_. Throughout surgery, sows received normal saline and Ringers solution (6–12 L given over 3–4 h, 4–15 mL/kg/h) and the following variables were monitored; O_2_ saturation (pulse oximetry), arterial blood pressure (Doppler or invasive via catheter), end-tidal isoflurane and end-tidal CO_2_ concentrations (Capnomac Anaesthesia Monitor; GE Heathcare, Chicage, IL).

Up to four piglets from each litter were resuscitated and maintained under neonatal intensive care conditions as previously described.^12^ All piglets remained in utero until a team was available for immediate resuscitation. Care was taken to prevent tension on the umbilical cord. Prior to cord clamping, blood samples were collected from the umbilical artery and vein, followed by administration of a propofol loading dose (0.5–0.75 mL; Provive MCT-LCT 1%; AFT Pharmaceuticals, NZ) through the umbilical vein to induce sedation. The cord was milked (5-7 times until minimal return) to ensure entire dose of propofol was administered and provide maximum blood volume possible at delivery. Piglets rarely gasped due to the respiratory depressant properties of maternal anaesthesia. Time of birth was recorded at clamping of the cord, 30–60 s after exteriorisation.

Preterm piglets were placed in open ended plastic sleeves to retain heat and reduce insensible water loss, then weighed and transferred to humidified isolettes within 30–60 s of delivery. Only piglets with a birth weight >10th and <90th percentile for their gestation were used. Birth weight centiles were calculated using our established cutoffs for preterm and term piglets (*n* > 1000). Isolettes were set between 36.5–39 °C and 60–80% humidity to maintain body temperature between 38 °C and 39.5 °C (normal body temperature for piglets). Piglets were intubated using a 2.5–3.0Fr (cuffed or uncuffed) endotracheal tube. Surfactant (2.5 mL/kg endotracheal; Curosurf, Chiesi, Australia) was administered to preterm piglets. All piglets were ventilated using a conventional neonatal ventilator, where fraction inspired oxygen (FiO_2_) was adjusted throughout the experiment to maintain a saturation target between 90% and 95%. Peak inspiratory pressure (PIP) was adjusted between 12–25mmH_2_O and breath rate was adjusted between 25 and 60bpm, with inspiration time set to 0.4 s to maintain a partial pressure for CO_2_ of 45–60 mmHg. Additional piglets were exsanguinated at delivery to collect plasma for laboratory standards then, along with the sow and littermates, were euthanised immediately after delivery of the experimental piglets. Experimental piglets were euthanised at the end of the 12 h period (phenobarbitone sodium 162 mg/kg; Virbac Australia; piglets: 0.5 mL IP, sow: 60 mL IV).

Piglets had venous umbilical catheters placed within 30 min of birth. Sedation and analgesia were maintained with a propofol-fentanyl mix (Provive 1%, Baxter Healthcare, AUS; Sublimaze; Janssen-Cilag, AUS). Sedation infusion was immediately commenced using a loading protocol where dosage was reduced across the first hour (term piglets: 20–4 mg/kg/h propofol and 4.50–0.89ug/kg/h fentanyl; preterm: 10–4 mg/kg/h propofol and 2.22–0.89ug/kg/h fentanyl). The target maintenance dose range (4–6 mg/kg/h propofol and 0.89-1.33 ug/kg/h fentanyl) was the same in term and preterm piglets. Due to their higher fat content, term piglets were typically at the upper end of this range. This maintenance dosage range was lower than a dose shown to have no adverse cardiovascular effects in piglets.^13^

Two umbilical artery catheters (UAC) were placed at different depths to minimise cross-contamination with dextran tracers; ~13 cm for administration of glucose (3 mL/kg/h, 10%; Baxter International, Deerfield, IL) and dextran administration and ~15 cm for continuous blood pressure monitoring and blood sample collection. Arterial blood gases analyses were conducted every 20-60 min throughout the experiment to monitor respiratory requirements (ABL815 Blood Gas Analyser; Radiometer, Denmark). The average maintenance fluids infused during the experiment, including glucose, arterial catheter fluid and sedation, was 5.5 ± 1.4 mL/kg/h for preterm piglets and 3.7 ± 0.7 mL/kg/h term piglets.

### Blood volume

Blood volume was measured at two timepoints in each piglet using two differently labelled 500 kDa dextrans. There were three different measurement time frames:

(a) 0.5 h and 5 h, *n* = 12 preterm piglets,

(b) 0.5 h and 9 h, *n* = 44 preterm and *n* = 40 term piglets,

(c) 5 h and 11 h, *n* = 7 preterm piglets.

Dextran-FITC or Dextran-TRITC (2 mg/mL; 1 mL in term piglets, 0.5 mL in preterm piglets) were administered via the glucose infusion line at the first and second timepoints, respectively. Blood samples (0.2 mL; serum separator tubes, SST) were collected at 5, 10, 15 and 20 min after dextran administration. Delays to catheter insertion resulted in 6 preterm and 4 term piglets having their first measurement >45 min after birth so these values were excluded.

Replacement transfusions were administered at 1, 2 and 3 h after the first volume measurement to replace blood losses due to sampling for both blood volume measurement and for blood gas analyses. The total volume of fresh whole sow blood (heparinised; 1000IU DBL heparin/mL blood; Hospira, Lake Forest, IL) given to replace sampling losses was similar between preterm and term piglets (4.0 ± 0.9 vs. 3.7 ± 0.4 mL; *P* = 0.24, *n* = 16/group). As sow haematocrit (Hct) averaged 0.29, lower than the average piglet Hct of 0.33, these replacement transfusions provided to an additional 0.16 mL and 0.15 mL of plasma in preterm and term piglets, respectively, above that required to replace plasma losses. Replacement transfusion with sow blood that had a lower haematocrit did not result in a decrease in piglet haemoglobin concentration.

To calculate blood volume, fluorescence intensity at time of injection was estimated using back extrapolation by fitting a straight line to the four timed samples. Standard curves of dextran concentrations were generated in plasma from littermates and used to convert fluorescence intensity to dextran concentration at time of injection. Fluorescence of duplicate plasma samples and standards (40 μL in 160 μL PBS) were measured (Spark 10 M multimode reader; Tecan, Switzerland). Plasma volume was calculated as: plasma volume = (mass of injected dextran)/(dextran concentration at time of injection).

Blood volume was calculated as: blood volume = (plasma volume)/(1-haematocrit).^14^ Blood volume was normalised to piglet mass and expressed as mL/kg.

### Plasma protein measurement

To determine plasma protein concentration and red cell indices (haematocrit, mean corpuscular volume and reticulocyte count), 0.5 mL arterial blood was collected alongside the 20 min sample at the time of each volume measurement (iDexx Vet Pathology, Brisbane, Australia).

### Statistical analysis

**Fig. 1:**
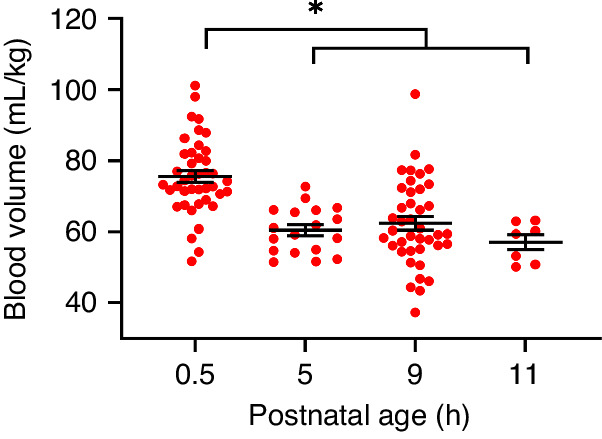
Rapid blood volume reducation in the hours after preterm birth. Blood volume in preterm piglets between baseline (31 ± 6 min) and 11 h postnatal age. *P < 0.001, indicates that blood volumes at 5–11 h postnatal are significantly lower than at 0.5 h. Values were similar at 5, 9 and 11 h postnatal age. Data is mean ± SEM.

**Fig. 2:**
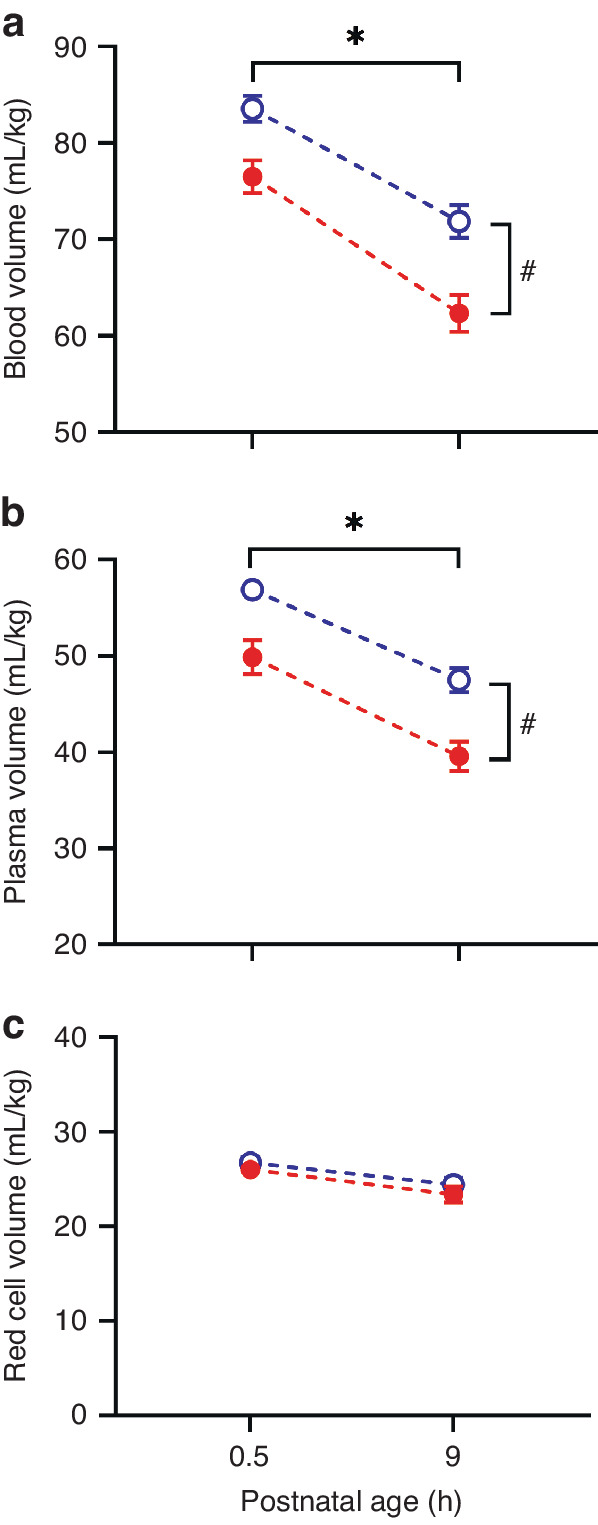
Blood and plasma volume reductions after birth. Changes in **a** blood volume (mL/kg), **b** plasma volume and **c** red cell volume between baseline (31 ± 6 min postnatal age) and 9 h postnatal age in term (open/blue circles, *n* = 40) and preterm (solid/red circles, *n* = 44) piglets. *indicates significant reduction in both groups between baseline and 9 h. ^#^Indicates that preterm piglets have a significantly lower volume than term piglets. Data is mean ± SEM.

**Fig. 3:**
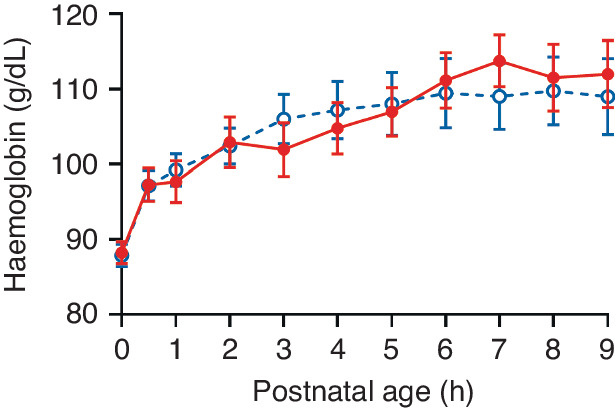
Haemoconcentration in the hours after birth. Haemoglobin concentration from birth (0 h: cord) to 9 h postnatal age in term (open/blue circles, *n* = 15) and preterm (solid/red circles, *n* = 15) piglets. Data is mean ± SEM.

**Fig. 4:**
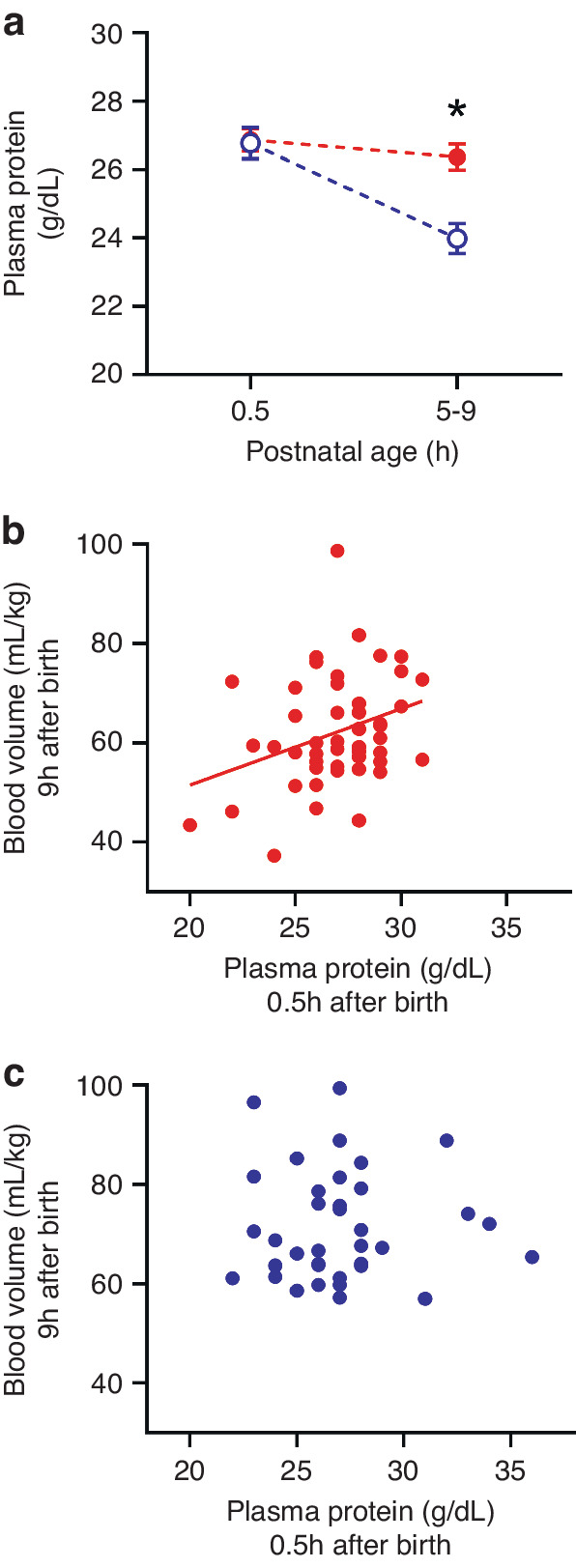
Low plasma protein predicts low blood volume in preterm piglets. **a** Change in plasma protein concentration between baseline (~30 min after birth) and 9 h postnatal age in preterm (solid red circles, *n* = 50) and term piglets (open blue circles, *n* = 36), and the relationships between plasma protein concentration at 0.5 h postnatal age and blood volume at 5–9 h in **b** preterm piglets (*R*^*2*^ = 0.10, *P* = 0.028, *n* = 50) and **c** term piglets (*R*^2^ < 0.001, *P* = 0.97, *n* = 36). *Indicates preterm piglets had significantly plasma protein levels at 5–9 h compared to at term.

All data were analysed using GraphPad Prism (Version 9.4.1, San Diego, CA) and IBM SPSS (Version 28.0.1.0). Data were tested for normality using skewness and kurtosis normality tests, with values between −2 and +2 considered acceptable. Data were normally distributed. Effects of postnatal age (Fig. 1) were analysed using one-way ANOVA and postnatal and gestational age differences (Figs. 2, 3 and 4a) were detected using 2-way repeated measures ANOVA with multiple comparisons tests. Associations between plasma protein concentration and blood volume were detected using Pearson’s correlation analyses (Fig. 4b, c). Data are reported as mean ± SD in tables and text and mean ± standard error of the mean (SEM) in figures. *P* < 0.05 was considered statistically significant.

## Results

A total of 103 piglets were included in this study and in each cohort, piglets are spread across at least four litters. Sex ratios and litter size were similar between groups (Table 1).

**Table 1:** Characteristics of term and preterm piglet cohorts.

	Term	Preterm		
	0–9 h	0–5 h	0–9 h	5–11 h
*n*	40	12	44	7
Litter size	13 (4)	13 (3)	13 (3)	12 (2)
Male:female	22:18	8:4	23:21	4:3
Body weight (g)	1516 (196)	994 (63)*	1039 (174)*	986 (45)*
PNA 0.5 h timepoint (min)	29 (4)	19 (7)	31 (7)	–

Data are mean (SD). *PNA* postnatal age. *Indicates all preterm chohorts were significantly lower than the term cohort (*p* < 0.0001).

Blood volume was highly variable in preterm piglets with a minimum of 52 ml/kg and maximum of 101 mL/kg (Fig. 1), this variability was particularly evident at 0.5 h after birth. Term piglets were also variable but to a lesser degree, with a minimum of 64 mL/kg and maximum of 99 mL/kg. In preterm piglets, blood volume was similar across the 5, 9 and 11 h postnatal ages (*P* > 0.92), and was significantly reduced by 5–11 h postnatal age (61 mL/kg (SD 10)) compared to 0.5 h (76 ml/kg (SD 11), *P* < 0.0001, 95% CI 10–26, Fig. 1).

At 0.5 h after birth, preterm piglets had significantly lower blood volumes than term piglets (76 (SD 10) *vs.* 84 (SD 8) mL/kg, *P* = 0.024, 95% CI 1–14, Fig. 2a). Comparison of the 0–9 h cohorts of preterm and term piglets, showed a significant reduction (*P* < 0.0001, 95% CI 10–16) in blood volume in both preterm (14 ml/kg (SD 11), 18% (SD 13)) and term piglets (11 mL/kg (SD 11), 13% (SD 12), Fig. 2a). At 9 h after birth, the average blood volume of preterm piglets (62 mL/kg (SD 12)) was 10 mL/kg or 14% less than in term piglets (72 mL/kg (SD 11), *P* = 0.0004, 95% CI 3–16, Fig. 2a). The magnitude of these blood volume reductions was similar between preterm and term cohorts (*P* = 0.25 for absolute and *P* = 0.074 for %change). There was no effect of birth order or time from start of caesarean surgery until delivery on blood volume (*P* > 0.2). No effect of sex on blood volume was detected (*P* = 0.29).

At 0.5 h and 9 h after birth, plasma volume was lower in preterm piglets than at term (*P* < 0.0001, 95% CI 5–10, Fig. 2b). Between 0.5 and 9 h, plasma volume significantly decreased by 11 mL/kg (SD 12) (34% (SD 18)) in preterm and 9 mL/kg (SD 8) (21% (SD 16) in term piglets (*P* < 0.0001, 95% CI 7–13, Fig. 2b). The absolute changes were similar between the groups (*P* = 0.49) whereas, the percentage change was greater in the preterm piglets than at term (*P* = 0.006, 95% CI 3–15). At 0.5 h and 9 h after birth, red cell volume was similar in preterm (26 (SD 5) and 23 (SD 5) mL/kg, respectively) and term piglets (27 (SD 4) and 24 (SD 5)mL/kg, respectviely) (*P* = 0.26). By 9 h, there were significant reductions in both preterm (3 mL/kg (SD 5)) and term piglets (2 mL/kg (SD4), both groups *P* = 0.02, 95% CI 1–4, Fig. 2c).

There was no difference in haemoglobin concentrations between term and preterm piglets across the 9 h after birth (*P* = 0.78, Fig. 3). Both groups had significant increases in haemoglobin levels between birth (cord) and 9 h (*P* < 0.0001, 95% CI: 11–34). Levels increased by 19% (SD 17) in preterm piglets and by 17% (SD 12) in term piglets, respectively. Haemoglobin levels increased most rapidly in the first 2 h after birth.

At 30 min after birth, plasma protein concentrations were similar in preterm and term piglets (*P* = 0.95). At 9 h after birth, levels had significantly decreased in preterm (*P* = 0.029) and term (*P* < 0.0001, 95% CI: 2.09–3.51) piglets, however values were significantly lower in term piglets (*P* = 0.0002, 95% CI: 1.03–3.60, Fig. 4a). There was a significant positive relationship between plasma protein levels at 30 min after birth and blood volume at 9 h in preterm (*R*^*2*^ = 0.10, *P* = 0.028, *n* = 50; Fig. 4b) but not term piglets (*R*^2^ < 0.001, *P* = 0.97, *n* = 36; Fig. 4c).

## Discussion

This study investigated changes in blood volume across the first hours of life in preterm and term piglets. Blood volume rapidly decreases in the hours after birth regardless of gestational age. In contrast to values reported in clinical resources, we show that blood volume is lower in preterm piglets compared with term piglets. Our data also show that preterm, but not term, piglets with low plasma protein concentration soon after birth are vulnerable to low blood volume after five hours postnatal age (Fig. 4).

### Preterms do not have a higher blood volume than terms

Neither preterm piglets nor preterm infants have a higher blood volume than their term counterparts, in contrast to current clinical resources which estimate a higher blood volume in preterm than term infants.^15,16^ Blood volume is 9 mL/kg (13%) lower in preterm piglets across the 5-9 h after birth than in term piglets. Similarly, some published values are 13% lower in very preterm infants than at term (71 vs. 82 mL/kg).^10,17,18^ and our recent review found no evidence for higher blood volumes in preterm infants.^19^ This is in contrast to clinical resources which give blood volume estimates for preterm infants that are higher than for term infants, for example 90 vs. 80 mL/kg.^15^ or 100 vs. 90 mL/kg.^16^ These higher volume estimates for preterm infants are based on early work by Sisson.^7^ using small tracers that are known to rapidly leak from the circulation, and therefore overestimate volume in more immature neonates.^7,20^

### Blood volume rapidly decreases in the first hours of life

The 24% reduction in blood volume that occurred between 0.5 h and 9 h after birth in preterm piglets is clinically very significant. Losses greater than 10% in adults and children are considered critical and typically volume expansion is given to prevent cardiovascular deterioration. Not only is this a substantial reduction but it occurs very rapidly after birth. Stable volumes were observed from 5 h after birth. However, the rapid early rise in haemoglobin concentration suggests that much of this loss occurs prior to 5 h after birth; indeed the most rapid change occurs within 2 h of birth. This reduction in blood volume appears to persist for at least 11 h. The extent and timing of blood volume reductions in preterm infants are unknown but early studies suggest a reduction in blood volume. Two studies of changes in blood volume over the first day in preterm infants report a 10% and 7% reduction in blood volume between 30 min and 4 h after birth.^5,6^ However, studies in preterm infants under modern care practices are lacking so our understanding of early blood volume changes in this population is very limited. Further studies of early blood volume and how it changes over the first day are essential to determine if similar reductions occur in preterm infants.

These rapid shifts in blood volume after birth may be part of the normal adaptation to ex utero life. However, in preterm infants this plasma shift may result in hypovolemia. The role of early hypovolemia in deterioration of cardiovascular function needs to be examined in preterm piglets and preterm infants. Term infants have relatively mature cardiovascular systems that are capable of compensating for changes in blood volume via vasoconstriction.^21^ The ability to constrict allows blood flow to be redirected to critical organs such as the brain. However, preterm infants are predisposed to vasodilation.^22–24^ and multiple pre-clinical studies demonstrate an immature capacity to vasoconstrict in response to stimuli.^22,25–27^ We have previously shown that preterm piglets were not able to maintain cerebral blood flow when blood volume is reduced. In contrast, term piglets were able to compensate for reduced blood volume and maintain cerebral blood flow.^28^ Preterm infants with even moderate blood volume reductions may be unable to maintain oxygen supply to the brain and other major organs, placing them at high risk of brain injury.

### Plasma loss from the circulation

These studies demonstrate that reductions in blood volume are due to plasma loss from the circulation rather than the loss of whole blood. In both piglet groups, plasma volume reduction accounted for the majority of the reduction in blood volume. This occurred in parallel to proportional increase in haemoglobin levels (19%) over the same period suggesting that plasma, and not whole blood, is leaking from the circulation. An early study in preterm infants also demonstrate an increase in haemoglobin levels of 9% between birth and 1–8 h after birth.^29^ Loss of whole plasma has also been implicated in term infants where injected iodine-labelled albumin (plasma tracer) levels decreased by 27.3% in the first 2.5 h of life.^30^ The alternative explanation, that haemoglobin levels increase due to the release of stored red cells, is not supported. The released reticulocytes (immature red cells) are larger than circulating cells and hence, mean corpuscular volume (average red cell size) would increase.^31^ In this study, the proportional increase in reticulocyte number was small, and mean corpuscular volume decreased, so the observed increase in haemoglobin levels was not driven by the release of cells. Indeed, there was a small decrease in red cell volume in preterm piglets. Our findings, coupled with clinical studies, provide strong evidence that observed reductions in blood volume are due to whole plasma loss from the circulation occurring rapidly in the few hours after birth.

### Preterm blood volume is highly variable

Blood volume is highly variable in both preterm piglets and preterm infants, ranging from 61101.^10^ This variability is higher than both term piglets or term infants, where term piglets ranged from 67 to 96 mL/kg and term infants ranged from 68 to 100 ml/kg.^10,32^ Preterm infants at the lower end of this range are likely to be hypovolemic. Variability in the contribution of multiple factors may be responsible for the wide range of measurements. Understanding of the various factors driving plasma loss in preterm neonates is essential to the development of effective blood volume support, both for prevention and more effective treatments.

There are multiple potential mechanisms that contribute to plasma leak in preterm piglets. Vascular immaturity coupled with a pro-inflammatory environment may result in greater capillary permeability in preterm piglets, allowing greater fluid movement and protein leak between the intravascular and extravascular space.^33^ Tissue pressure decreases in the transition to *ex utero* life, due to loss of intraamniotic pressure,^34^ may promote plasma leak from the circulation. Lymphatic flow returns lost fluid back into circulation and is very high *in utero* preventing net fluid loss.^35^ If lymphatic flow rate is reduced after birth, it may be inadequate to match excessive plasma loss from the circulation, and thus may contribute to the decrease in blood volume.^36^ Most infants do not develop severe oedema but the preterm infant is known to have a massive transdermal water loss during this time.^37^ so significant fluid may be lost from the extravascular space. Further preclinical and clinical studies are necessary to fully understand mediators driving plasma loss and blood volume changes in infants.

Increases in blood volume at birth are rapidly lost in the hours after birth so may not protect against hypovolemia. Placental transfusions and cord milking provide comparable additional blood volumes at birth.^38^ To best mimic the clinical practice of delayed cord clamping, all piglets received umbilical cord milking as short umbilical cords and large maternal size do not allow for piglets to be positioned for placental transfusion. Delayed cord clamping does increase blood volume at birth in both term and preterm infants.^11,39^ However, this additional plasma is lost within hours such that infants that had early or delayed cord clamping had similar plasma volumes by 2 h after birth.^11,39^ Similarly, in this study blood volume reduction within hours of birth was due to plasma loss despite all receiving umbilical cord milking. Also modern clinical care encourages respiratory effort prior to cord clamping whereas in this study there was no respiratory effort in term or preterm piglets before cord clamping. This may have impacted the volume of placental transfusion however, all piglets received the same care and preterm piglets still have a lower blood volume than at term.

### Low plasma proteins and low blood volume in preterm piglets

Low plasma protein concentration may be an important driver of blood volume reduction in preterm neonates. Preterm piglets with lower plasma protein concentration at birth were more likely to have low blood volume at 5–9 h postnatal age. Whilst the relationship between plasma protein and infant blood volume has not been well-characterised, preterm infants with hypoproteinaemia (<40 mg/mL) have an increased incidence of hypotension.^40^ and long term neurological injury.^41,42^ One explanation for this may be that low plasma protein concentration favours plasma loss by reducing the oncotic pressure gradient across the capillary wall, which is maintained by the difference in protein concentration between the vascular and extravascular space. The oncotic pressure gradient is a major driving force opposing fluid loss from the circulation, as described in the Starling Equation.^30,43,44^ It should be noted however that we and others measured piglet plasma protein to be approximately 24 g/dL in both term and preterm piglets.^45^ In contrast, preterm infants have plasma protein concentrations substantially lower than at term (39 vs. 68 g/L),^46^ suggesting that preterm infants may be at greater risk of plasma loss due to a reduced capacity to generate an oncotic pressure gradient across the capillary wall. Further studies are warranted to understand the role of plasma proteins, along with other mediators driving plasma loss and blood volume changes such as inflammation and lymphatic return.

### Limitations

We were not able to measure blood volume at birth and thus it is possible that our data underestimates the blood volume change. Haemoglobin levels from cord sample suggests that plasma volume was substantially larger at birth than at the baseline measurement and decreased most rapidly in the first 30 min of life, Thus, much of the volume decrease may not have been detected by this study. Additionally, our preclinical model may not emulate the increased blood volume that would be expected from cord clamping occurring after respiratory effort. However, clinical data and this study provide strong evidence that rapid blood volume changes occur after birth and that preterm blood volume is highly variable and lower than at term.

## Conclusion

In contrast to clinical resources, preterm piglets have a lower blood volume than at term. There is a substantial and rapid reduction in blood volume after birth regardless of gestational age. This reduction may be part of normal adaptation to ex utero life. However, as preterm newborns have limited ability to vasoconstrict and hence compensate for this reduction in blood volume, this may contribute to the risk of cardiovascular instability. This study highlights the need to understand early changes in blood volume in preterm infants, its drivers and consequences.

### Data accessibility

The datasets generated during and analysed during the current study are available from the corresponding author on reasonable request.

## Data Availability

The datasets generated during and analysed during the current study are available from the corresponding author on reasonable request.
